# High insecticide resistances levels in *Anopheles gambiaes* s.l. in northern Uganda and its relevance for future malaria control

**DOI:** 10.1186/s13104-020-05193-0

**Published:** 2020-07-22

**Authors:** Richard Echodu, Julius Iga, William Samuel Oyet, Paul Mireji, Juliet Anena, David Onanyang, Tereza Iwiru, Julius Julian Lutwama, Elizabeth Auma Opiyo

**Affiliations:** 1 Department of Biology, Faculty of Science, Gulu University, P.O. Box 166, Gulu, Uganda; 2 Gulu University Biosciences Research Laboratories, P.O. Box 166, Gulu, Uganda; 3 Department of Biochemistry, Biotechnology Research Institute-Kenya Agricultural and Livestock Research Organization, Kikuyu, Kenya; 4 Department of Arbovirology, Uganda Virus Research Institute, Entebbe, Uganda

**Keywords:** Pyrethroid resistance, Malathion, Bendiocarb, Deltamethrin and permethrin, Uganda

## Abstract

**Objective:**

The aim of the study was to determine the level of insecticide resistance and diversity in *Anopheles* mosquitoes in northern Uganda. Standard WHO insecticide susceptibility test assays were used to test for susceptibility to 0.5% malathion, 0.1% bendiocarb, 0.05% deltamethrin and 0.75% permethrin on 3–5 day old generation one progeny. We also screened for species diversity and knockdown resistance using PCR assay.

**Results:**

*Anopheles gambiae s.s.* is the predominant malaria vector in northern Uganda followed by *An. arabiensis. An. gambiae* s*.s.* was susceptible to malathion and bendiocarb with the observed mortality rate of 100% and 98–100% observed respectively while very high resistance was observed with deltamethrin and permethrin. Minimal KDR-eastern variant homozygous forms of 8.3% in *An. gambiae s.s.* were detected in Oyam district. In conclusion, this study confirms that *An. gambiae s.s.* females are susceptible to malathion and bendiocarb while high intensity of resistance was observed with deltamethrin and permethrin in the same area. Use of carbamate and organophosphate insecticides bendiocarb and malathion for indoor residual spraying activities in northern Uganda is highly recommended since high levels of pyrethroids resistance (deltamethrin and permethrin) was detected in the area.

## Introduction

Uganda is ranked fourth among the 15 high-burden countries that carry 80% of the global malaria burden [1]. Malaria remains the number one leading cause of morbidity and mortality in the country with an estimated 16 million cases and over 10,500 deaths per year, respectively [2]. In northern Uganda particularly, the 26 years of civil war in the region, beginning in the mid-1980s and lasting until 2006 [3, 4] pushed the area to register the highest number of malaria cases recorded in the country (63% prevalence in 2009) [2, 5, 6].That being said, the region also has a long history of IRS and LLINs usage for management of malaria vectors [7]. Household ownership of at least one insecticide treated bed net in the region stood at 81.8% in 2010 [8]. Worldwide, the current use of LLINs and IRS for effective malaria vector control is challenged by widespread insecticide resistance in mosquito populations [9, 10]. There is also heavy usage of pesticides for agricultural purposes in northern Uganda [11, 12]. The use of pesticides for agricultural purposes are known to contribute to selection of resistance in mosquitoes which undermines malaria vector control interventions [13]. All these factors could be driving mosquito’s resistances to insecticides in northern Uganda.

Insecticide resistance monitoring and surveillance is highly recommended by World Health Organization (WHO) for guiding national monitoring and management plans [14]. In northern Uganda, despite having the highest malaria prevalence rate in the country, little attention has been paid to the contribution of mosquito resistance to high malaria prevalence seen in the region. The use of preventive intervention measures like IRS and LLINs are known to impact on the resting and feeding behavior of major malaria vectors [15] and can result in shift in mosquito species compositions [16, 17]. This happens as a result of the more exophagic, exophilic and zoophilic nature of *An. arabiensis* compared to *An. gambiae s.s.* and *An. funestus* behaviors which can similarly affect the performance of IRS. It has been reported in Kenya and Tanzania that vector control intervention might be less effective against *An. arabiensis*, that is less killed by LLIN and treated with pyrethroids as compared to their counterparts *An. gambiae s.s.* and *An. funestus* [18, 19]. We investigated insecticide susceptibility status and diversity of malaria vectors in northern Uganda. This information may be used in planning future malaria control programmes and for efficient management of insecticide resistance strategies in the study area.

## Main text

### Methods

#### Study sites

This study was carried out in districts of Kitgum (3°17′ 20.0″ N, 32°0.52′ 40.0″ E), Lamwo (3°32′ 0″ N, 32°48′ 0″ E), Agago (2°49′ 59″ N, 33°19′ 60″ E), Gulu (2°44′ 59″ N, 32°00′ 0″ E), Oyam (2°22′ 52″ N, 32°30′ 2″ E) and Pader (2°49′ 59″ N, 33°19′ 60″ E) during the rainy season of 2017, 2018 and the dry season of February 2019 [20, 21].

#### Collection of adult and larval mosquitoes

Collection of adult *Anopheles* mosquitoes took place once during the rainy season in May of 2017, in April, June–September of 2018 and during the dry season in February of 2019. Two sub-counties from each district were randomly selected out of which two villages were chosen at random for study (Table S1). Collection of indoor resting adult mosquitoes in households was done between 6 a.m. and 12 noon using pyrethrum spray collection (PSC) method [22] and identified to the species level according to Gilles [23]. Collection of *An. gambiae* s.l. larvae were done independently from adult in sand mining pits, quarry, brick making sites, river beds and rain water collection sites.

**Table 1:** Median Lethal Time for knockdown in Adult female *An. gambaie s.s.* mosquitoes (from Gulu district, Uganda) by various insecticides

Insecticide	LT_50_, Min	95% CI	Slope (β ± SE)	χ^2^
Deltamethrin	104.9	88.30–134.4	1.302 ± 2.078	1.621
Permethrin	0	Very wide	Very wide	7.348
Bendiocarb	22.73	21.92–23.57	3.564 ± 4.488	1.926
Malathion	19.62	17.84–21.53	3.504 ± 8.212	5.692

#### Insecticide susceptibility bioassay tests

3–5 day old F1 female progeny mosquitoes were randomly selected and subjected to standard WHO susceptibility tests [24]. We exposed a total 400 female *An. gambiae* s.l. to different standard WHO insecticide-treated papers having discriminating susceptibility dosage of 0.1% bendiocarb, 0.75% permethrin, 0.05% deltamethrin and 0.5% malathion to assess potential insecticide resistance. Kisumu strain of *An. gambiae s.s.* were used as negative control. Knockdowns time of 10, 15, 20, 30, 40 min through to 60 min after the start of exposure were recorded. Mortality was recorded 24 h after exposure.

#### Detection of East African KDR resistance mutations in *An. gambiae* s.l

We used knockdown resistance method described by Ranson [25] to assay for single base pair specific single nucleotide polymorphisms (SNPs) leucine to serine substition TTA/TCA mutation in the voltage-gated sodium channel. We amplified 5 µl gDNA extracted using Qiagen kit (Qiagen, Valencia, CA, USA), 0.2 μM of the specific primers (AgD1, AgD2, AgD4 and AgD5) with 1 unit of GoTaq Green Master Mix (Promega, Madison, MO) in the buffer in a total volume of 25 µl using PCR (SimpliAmp, Applied Biosystems, Life Technologies, Singapore). PCR methods described by Scott [26] for *An. gambaie* s.l. and for members of the *An. funestus* s.l. group by Koekemoer [27] were used for mosquitoes species identification.

#### Statistical analysis

We applied the Abbott’s formula to correct the knockdown rates for testing the toxicity of each insecticide [28] and then transformed them to Probits [29] for linear regression analysis and the determination of 50% knockdown (KDT_50_). For Probit analysis, we used GraphPad Prism version 7.00 for Mac (La Jolla, CA, USA). We used weighted mean to summarize knockdown due to different insecticides, and adopted the WHO criteria [24] to interpret our results.

#### Ethical consideration

Permission to conduct the study was granted by the Uganda National Council for Science and Technology and the Office of the Ugandan President (SS4610). Heads of households provided written informed consent.

### Results

#### Distribution and seasonal variation in adult *Anopheles* mosquito populations

More *Anopheles* mosquitoes were collected in Oyam followed by Gulu, Agago, Kitgum, Lamwo and Pader (Additional file 1: Table S1). There was a noted seasonal variation in the mosquito collection (Additional file 1: Table S1).

Out of the 270 adult mosquitoes collected by PSC and analyzed by PCR, 8% (22/270) were *An. arabiensis* and 92% (248/270) *An. gambiae s.s.* with varied distribution across the six districts (Additional file 2: Table S2). No *An. funestus* was detected.

#### Susceptibility of *An. gambiae* s.l. to insecticides

**Fig. 1:**
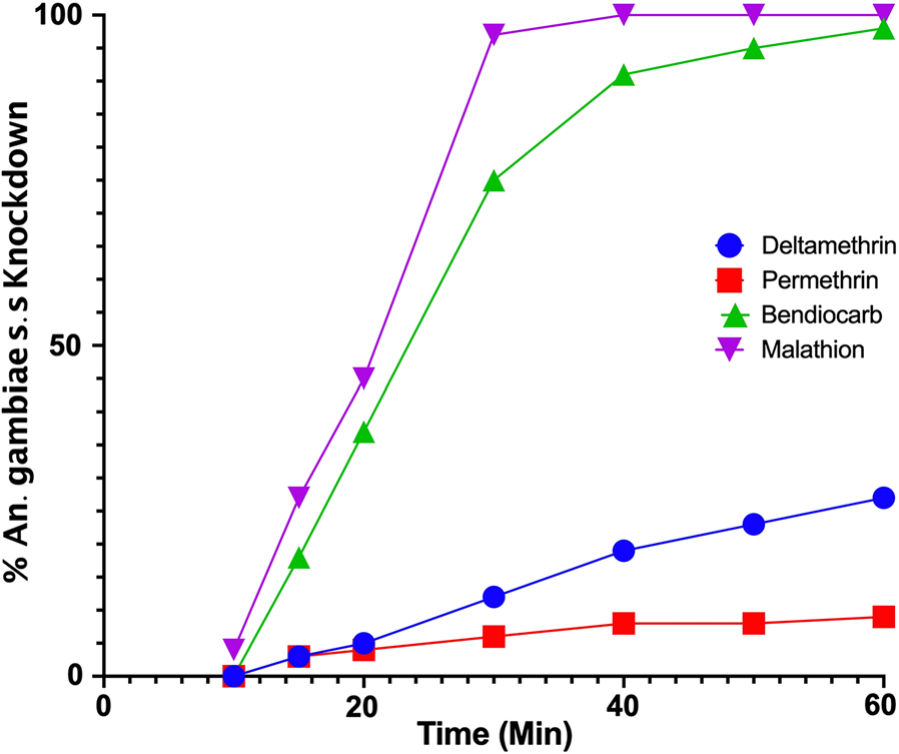
Percentage of *An. gambiae* s.l. knocked down during 60-min exposure to bendiocarb, deltamethrin, permethrin and malathion using the WHO tube assay in Gulu District in Uganda, November, 2018 (larval collections)

It was not always possible to bioassay the recommended number of mosquitoes (i.e., 100 specimens per location in Agago, Oyam and Kitgum) due to the low density of mosquitoes collected in the surveys. Susceptibility of *An. gambiae* s.l. to bendiocarb and malathion was observed in Gulu with mortality rate of 98% and 100% (Fig. 1, Additional file 3: Table S3). Resistance (< 95% mortality) of *An. gambiae* s.l. to permethrin and deltamethrin was observed in all the samples with mortality varying from 5% to 6% in Gulu (Table S4). The LT_50_ for malathion and bendiocarb on *An. gambiae s.s.* were shorter compared to deltamethrin and permethrin (Table 1).

Our subsequent PCR of sibling species on most of the mosquitoes (N = 455) post-exposure revealed that all these mosquitoes to be *An. gambiae s.s.* (100%).

#### Prevalence of East African (L1014S) knockdown resistance (KDR) point mutations in *An. gambiae s.s.* in Gulu district

The summary of L1014S mutations from genotyping of *An. gambiae s.s.* (n = 159) from PSC collection and *An. gambiae s.s.* (n = 400) from susceptibility test assays are in Additional file 2: Table S2 and Additional file 4: Table S4. Most of the PSC and susceptibility tested *An. gambiae s.s.* mosquitoes were homozygous for susceptible wild type (Table 2 and Additional file 2: Table S2).

**Table 2:** KDR allele frequencies

District	Mosquitoes species	Insecticide	Survival status after exposure	# mosquitoes tested	# No amplification samples	Homozygote mutation (RR)	Homozygote wild type (SS)
Gulu	*An. gambiae s.s.*	Permethrin 0.75%	Dead	12		8	4
Gulu	*An. gambiae s.s.*	Permethrin 0.75%	Live	100	16	57	27
Gulu	*An. gambiae s.s.*	Malathion 0.5%	Dead	99	21	59	18
Gulu	*An. gambiae s.s.*	Deltamethrin 0.05%	Live	21	10	7	4
Gulu	*An. gambiae s.s.*	Bendiocarb 0.1%	Live	2	1	1	
Gulu	*An. gambiae s.s.*	Bendiocarb 0.1%	Dead	95	18	49	28
Gulu	*An. gambiae s.s.*	OP/C control	Live	34	2	30	2

### Discussions

We report high level of insecticides resistance to the commonly used pyrethroids insecticides permethrin and deltamethrin in the region. This could be due to the long-term use of pyrethroids insecticides for IRS and pyrethroid-treated LLINs in the region. For instance since 1960 there were no IRS activities in region until 2005 when it was introduced as a result of malaria epidemics in refugee camps [7, 30] From 2007 to 2009 spraying was done biannually using pyrethroid insecticide, alpha-cypermethrin but in 2010, it was shifted to a carbamate insecticide, bendiocarb due to high mosquito resistance [7, 31].

What is clear from these results is that the current use of pyrethroids (permethrin, deltamethrin) in northern Uganda is less effective against malaria vectors due to resistance of these mosquitoes to pyrethroid insecticides. Similar studies conducted in 2009 showed resistances to both DDT and the pyrethroids l-cyhalothrin, permethrin, lambdacyhalothrin in all places in northern Uganda [31, 32]. What could still be fueling this continued high level of resistance to pyrethroids seen here in could be the selection pressure attributed to long term history of usage of IRS and LLINs in the management of malaria vectors in the region. A similar study conducted by Bawuba [7] suggests selection pressure to pyrethroids as one factor. The selection pressure could also be attributed to use of similar classes of insecticides in agriculture [33]. It is also worth noting that pyrethroid resistance of malaria vectors is not a Ugandan problem alone, but a widespread problem across Africa [9, 24, 34].

Our results showed high number of *An. gambiae s.s.* mosquitoes collected during dry season as compared to rainy season in Gulu and Kitgum. This indicates that IRS and LLINs is not having impact on the species composition in northern Uganda. It also shows that the withdrawal of IRS which was done in 2014 stabilized mosquito population sizes and their distribution in the region. *An. gambiae s.s.* has been and still is the predominant indoor resisting vector in the region. Previous studies identify *An. gambiae s.s.* as the major malaria vector in northern Uganda [31, 35]. However, the ability of these vectors to predominate during dry season could possibly be explained by grass thatched housing structures that provide cool conditions for habitation of mosquitoes. This has a far reaching implication in the transmission of the malaria in the area and it could explain why there is constant malaria transmission throughout the year. Besides, we also see *An. arabiensis* in low density in all the six districts and still contribute to malaria transmission.

#### Implication for future malaria vector control

High level of resistance currently seen in malaria vectors in northern Uganda reduces the efficacy of pyrethroid-based interventions in the region. Since bendiocarb and malathion insecticides are still showing effectiveness for malaria vector-control programmes, the two insecticides should be used in rotation and sequenced with other insecticides that have different mode of action. The inclusion of *An. arabiensis* in the study is a call for incorporating other malaria vector control interventions that target outdoor biters’ into future studies.

### Conclusions

*An. gambiae s.s* is the predominant malaria vector followed by *An. arabiensis. An. gambiae* s.l. females in northern Uganda are still susceptible to malathion and bendiocarb while high level of resistance was seen in deltamethrin and permethrin. Metabolic phenotype resistance seem to contribute to the pyrethroid resistance with little genetic (KDR) mutations for resistance.

## Limitations

We acknowledge the limitations of the current study including:

- Time constraints and limited mosquito larval samples prevented us from conducting more susceptibility tests.

## Supplementary information

**Additional file 1: Table S1.** Seasonal comparison of female *Anopheles* mosquito population distribution.

**Additional file 2: Table S2.** Seasonal variation in *Anopheles* and their KDR distribution.

**Additional file 3: Table S3.** Knockdown summary during 60 min exposure (KD60).

**Additional file 4: Table S4.** Percentage mortality (24 h) of *An. gambiae s.l.* after 60-min exposure (KD60) to bendiocarb, permethrin, deltamethrin and malathion.

## Data Availability

The authors declare that all the main data supporting the findings of this study are available within the article (and its supplementary information files).

## Abbreviations

IRS: Indoor residual spraying
LLINs: Long lasting insecticide-treated nets
WHO: World Health Organization
KDR: Knockdown resistance
PCR: Polymerase chain reaction assay
PSC: Pyrethrum spray collection method
SSA: sub-Saharan Africa
ACT: Artemisinin-based combination therapy
PMI: President’s Malaria Initiative
IPTP: Intermittent preventive treatment
KDT50: Knockdown time
ITN: Insecticide-treated nets
LRA: Lord’s Resistance Army
IDP: Internally displaced person
GPS: Global Positioning System
SNPs: Single nucleotide polymorphisms

## References

[1] WHO, World Malaria Report 2017, 2017. 10.1071/ec12504.

[2] Ministry of Health (Uganda), Uganda malaria reduction strategic plan 2014–2020, Uganda Minist. Heal. (2014) 1–83.

[3] Behrend H, James O, Martinez I (1999). Alice Lakwena & the Holy Spirits : War in Northern Uganda 1986–97.

[4] C. Dolan, Social Torture: The Case of Northern Uganda, 1986-2006, ISBN 978-1-84545-565-1. (2009) 338. 10.1017/cbo9781107415324.004.

[5] Proietti C, Pettinato DD, Kanoi BN, Ntege E, Crisanti A, Riley EM, Egwang TG, Drakeley C, Bousema T (2011). Continuing intense malaria transmission in northern Uganda. Am J Trop Med Hyg.

[6] Okullo AE, Matovu JKB, Ario AR, Opigo J, Wanzira H, Oguttu DW, Kalyango JN (2017). Malaria incidence among children less than 5 years during and after cessation of indoor residual spraying in Northern Uganda. Malar J.

[7] Tukei BB, Beke A, Lamadrid-Figueroa H (2017). Assessing the effect of indoor residual spraying (IRS) on malaria morbidity in Northern Uganda: a before and after study. Malar J.

[8] U.M.S. Project, Malaria Intervention Coverage and Associated Morbidity Survey in Children Under Five Years: Indoor Residual Spraying in Northern Uganda and LLIN Coverage in Central Uganda. Kampala, Uganda., Uganda Malar. Surveill. Proj. (2012) 2012.

[9] Ranson H, N’Guessan R, Lines J, Moiroux N, Nkuni Z, Corbel V (2011). Pyrethroid resistance in African anopheline mosquitoes: What are the implications for malaria control?. Trends Parasitol.

[10] Strode C, Donegan S, Garner P, Enayati AA, Hemingway J (2014). The impact of pyrethroid resistance on the efficacy of insecticide-treated bed nets against African Anopheline Mosquitoes: systematic review and meta-analysis. PLoS Med.

[11] C A Omongo, E Adipala M. W Ogenga-Latigo, S. Kyamanywa, Insecticide application to reduce pest infestation and damage on cowpea in Uganda, African Plant Prot. 4 (1998) 91–100.

[12] L. Bategeka, J. Kiiza, I. Kasirye, Institutional Constraints to Agriculture Development in Uganda, Res. Ser. (2013) 1–44.

[13] Reid MC, McKenzie FE (2016). The contribution of agricultural insecticide use to increasing insecticide resistance in African malaria vectors. Malar J.

[14] World Health Organization, World malaria report 2018, 2018. www.who.int/malaria%0Ahttps://apps.who.int/iris/bitstream/handle/10665/275867/9789241565653-eng.pdf?ua=1%0A.

[15] B.B. Gillies, M. T. ; De Meillon, The Anophelinae of Africa south of the Sahara (Ethiopian zoogeographical region)., Anophelinae Africa South Sahara (Ethiopian Zoogeographical Reg. 343 (1968).

[16] Bayoh MN, Mathias DK, Odiere MR, Mutuku FM, Kamau L, Gimnig JE, Vulule JM, Hawley WA, Hamel MJ, Walker ED (2010). Anopheles gambiae: historical population decline associated with regional distribution of insecticide-treated bed nets in western Nyanza Province, Kenya. Malar J.

[17] Reddy MR, Overgaard HJ, Abaga S, Reddy VP, Caccone A, Kiszewski AE, Slotman MA (2011). Outdoor host seeking behaviour of Anopheles gambiae mosquitoes following initiation of malaria vector control on Bioko Island, Equatorial Guinea. Malar J.

[18] Kitau J, Oxborough RM, Tungu PK, Matowo J, Malima RC, Magesa SM, Bruce J, Mosha FW, Rowland MW (2012). Species shifts in the anopheles gambiae complex: do LLINs successfully control anopheles arabiensis?. PLoS ONE.

[19] McCann RS, Ochomo E, Bayoh MN, Vulule JM, Hamel MJ, Gimnig JE, Hawley WA, Walker ED (2014). Reemergence of Anopheles funestus as a vector of *Plasmodium falciparum* in Western Kenya after long-term implementation of insecticide-treated bed nets. Am J Trop Med Hyg.

[20] R.S. Twinomujuni NK, Sempagala-Mpagi RA, Uganda districts information handbook, Kampala Fountain Publ. (2011) 372.

[21] Uganda Bureau of Statistics, National Population and Housing Census 2014, Uganda Bur. Stat. 2014

[22] World Health Organization., Manual on practical entomology in malaria, World Heal. Organ. Geneva, Switz. (1975) 1975.

[23] M. Gillies, M.T. and Coetzee, A supplement to the Anophelinae of Africa South of the Sahara (Afro-Tropical region), South African Inst. Med. Res. Johannesburg. 55 (1987).

[24] WHO/GMP, Test procedures for insecticide resistance monitoring in malaria vector mosquitoes: Second edition, World Heal. Organ. Tech. Rep. Ser. (2016) 1–54. https://www.who.int/malaria/publications/atoz/9789241511575/en/.

[25] Ranson H, Jensen B, Vulule JM, Wang X, Hemingway J, Collins FH, Dame N, Biology V (2000). Identification of a point mutation in the voltage-gated sodium channel gene of Kenyan *Anopheles gambiae* associated with resistance to DDT and pyrethroids. Insect Mol Biol.

[26] Scott JA, Brogdon WG, Collins FH (1993). Identification of single specimens of the Anopheles gambiae complex by the polymerase chain reaction. Am J Trop Med Hyg.

[27] Koekemoer LL, Kamau L, Hunt RH, Coetzee M (2002). A cocktail polymerase chain reaction assay to identify members of the Anopheles funestus (Diptera: Culicidae) group. Am J Trop Med Hyg.

[28] Busvine J (1971). R, A Critical Review of the Techniques for Testing Insecticides. Commonw. Inst. Entomol. London..

[29] Finney, Probit Analysis, NCSS Stat. Softw. 575 (1971) 1–7. doi:10.1002/9781119407201.ch9

[30] National Malaria Control Programm, An Epidemiological Profile of Malaria and its Control in Uganda, Uganda Minist. Heal. (2013) 1–178.

[31] Okia M, Hoel DF, Kirunda J, Rwakimari JB, Mpeka B, Ambayo D, Price A, Oguttu DW, Okui AP, Govere J (2018). Insecticide resistance status of the malaria mosquitoes: *Anopheles gambiae* and *Anopheles funestus* in eastern and northern Uganda. Malar J.

[32] M. Okia, R. Ndyomugyenyi, J. Kirunda, A. Byaruhanga, S. Adibaku, D.K. Lwamafa, F. Kironde, Bioefficacy of long-lasting insecticidal nets against pyrethroid-resistant populations of Anopheles gambiae s.s. from different malaria transmission zones in Uganda, Parasites and Vectors. 6 (2013) 1–10. 10.1186/1756-3305-6-130. 10.1186/1756-3305-6-130 PMC3656772 23634798

[33] Maharaj R (2011). Global trends in insecticide resistance and impact on disease vector control measures. Insect Physiol.

[34] N’Guessan R, Corbel V, Akogbéto M, Rowland M (2007). Reduced efficacy of insecticide-treated nets and indoor residual spraying for malaria control in pyrethroid resistance area, Benin. Emerg Infect Dis.

[35] Okello PE (2006). Wim Van Bortel, Anatol Maranda Byaruhanga, Anne Correyn, Patricia Roelants, Ambrose Talisuna, Umerto D’alessandro, Variation in malaria Transmission intensity in seven sites thorughout Uganda. Am J Trop Med Hyg.

