# Supplementary material for: High insecticide resistances levels in Anopheles gambiaes s.l. in northern Uganda and its relevance for future malaria control
Source: BMC Res Notes. 2020 Jul 22;13:348. doi: 10.1186/s13104-020-05193-0 (PMC7376877; doi:10.1186/s13104-020-05193-0)
Supplement: Supplementary file 1 — Additional file 1: Table S1. Seasonal comparison of female Anopheles mosquito population distribution. [file 13104_2020_5193_MOESM1_ESM.docx]

**Appendix 1: Additional files**

**Table S1.** Seasonal comparison of female *Anopheles* mosquito population distribution.

| **District** | **Sub county** | **Parish** | **Village** | **No. of households visited** | **Total *Anopheles* mosquitoes collected** | |
| --- | --- | --- | --- | --- | --- | --- |
|  |  |  |  |  | **Rainy Season** | **Dry Season** |
| Gulu | Unyama | Pakwelo | Akonyibedo | 12 | 6 | 13 |
|  |  |  | Ajuku | 12 | 1 | 9 |
|  | Awach | Gwengdiya | Pegeya | 12 | 2 | 18 |
|  |  |  | Pugenyi | 12 | 1 | 33 |
| Oyam | Acaba | Abanya | Motmotatwero | 12 | 10 | 13 |
|  |  |  | Barowor | 12 | 26 | 9 |
|  | Minakulu | Adel | Obapo | 12 | 10 | 18 |
|  |  | Aceno | Bungiping | 12 | 94 | 33 |
| Kitgum | Layamo | Pagen | Lelamur | 12 | 0 | 13 |
|  |  |  | Gwengajut | 12 | 3 | 9 |
|  | Kitgum matidi | Oryang B’ | Putuke | 12 | 2 | 18 |
|  |  |  | Punu Col | 12 | 5 | 33 |
| Agago | Agago T/C a | Agago Central | Anyena | 12 | 3 | 0 |
|  |  |  | Olworguu | 12 | 4 | 0 |
|  | Parabongo | Pacer | Yot Kom | 12 | 9 | 12 |
|  |  |  | Jinja | 12 | 41 | 3 |
| Lamwo | Padibe West |  | Ram Ram | 12 | 5 | - |
|  | Padibe T/C a |  | Kamama Central | 12 | 1 |  |
| Pader | Ogom |  | Telela Central | 12 | 1 |  |
|  | Ogom |  | Owaleng Valley | 12 | 1 |  |
| **Total** |  |  |  | **240** | **225** | **234** |

a T/C: Trading Center
