# Supplementary material for: High insecticide resistances levels in Anopheles gambiaes s.l. in northern Uganda and its relevance for future malaria control
Source: BMC Res Notes. 2020 Jul 22;13:348. doi: 10.1186/s13104-020-05193-0 (PMC7376877; doi:10.1186/s13104-020-05193-0)
Supplement: Supplementary file 2 — Additional file 2: Table S2. Seasonal variation in Anopheles and their KDR distribution. [file 13104_2020_5193_MOESM2_ESM.docx]

**Table S2: Seasonal variation in *Anopheles* and their KDR distribution**

| **District** | **Sub county** | **Village** | **Season** | **Mosquitoes species** | **# Mosquitoes tested** | **# No amplification samples** | **Homozygote mutation (RR)** | **Homozygote wild type (SS)** | **Resistance status** |
| --- | --- | --- | --- | --- | --- | --- | --- | --- | --- |
| Agago | Parabongo | Jinja | Dry season | *An. arabiensis* | 1 |  |  | 1 | Susceptible |
| Agago | Parabongo | Jinja | Dry season | *An. gambiae s.s.* | 2 |  |  | 2 | Susceptible |
| Agago | Parabongo | Jinja | Rainy season | *An. arabiensis* | 4 | 1 |  | 3 | Susceptible |
| Agago | Parabongo | Jinja | Rainy season | *An. gambiae s.s.* | 35 | 13 |  | 25 | Susceptible |
| Agago | Agago Trading Centre | Olworguu | Rainy season | *An. arabiensis* | 1 | 1 |  |  |  |
| Agago | Agago Trading Centre | Olworguu | Rainy season | *An. gambiae s.s.* | 6 | 5 |  | 1 | Susceptible |
| Agago | Parabongo | Yot Kom | Dry season | *An. gambiae s.s.* | 6 | 6 |  |  |  |
| Agago | Parabongo | Yot Kom | Rainy season | *An. arabiensis* | 3 | 2 |  | 1 | Susceptible |
| Agago | Parabongo | Yot Kom | Rainy season | *An. gambiae s.s.* | 9 | 6 |  | 3 | Susceptible |
| Gulu | Unyama | Ajuku | Dry season | *An. gambiae s.s.* | 4 | 3 |  | 1 | Susceptible |
| Gulu | Unyama | Akonyibedo | Dry season | *An. gambiae s.s.* | 1 | 1 |  |  |  |
| Gulu | Unyama | Akonyibedo | Rainy season | *An. gambiae s.s.* | 2 | 1 |  | 1 | Susceptible |
| Gulu | Awach | Pageya | Rainy season | *An. arabiensis* | 1 | 1 |  |  | Susceptible |
| Gulu | Awach | Pageya | Rainy season | *An. gambiae s.s.* | 10 | 3 |  | 7 | Susceptible |
| Kitgum | Kitgum Matidi | Punu col | Dry season | *An. gambiae s.s.* | 5 | 3 |  | 2 | Susceptible |
| Kitgum | Layamo | Lelamur | Rainy season | *An. gambiae s.s.* | 1 | 1 |  |  |  |
| Kitgum | Kitgum Matidi | Putuke East | Dry season | *An. gambiae s.s.* | 8 | 6 |  | 2 | Susceptible |
| Lamwo | Padibe West | Ram Ram | Rainy season | *An. gambiae s.s.* | 5 | 4 |  | 1 | Susceptible |
| Lamwo | Padibe Trading Centre | Kamama Central | Rainy season | *An. gambiae s.s.* | 1 | 1 |  |  |  |
| Oyam | Acaba | Barowor | Dry season | *An. gambiae s.s.* | 1 |  |  | 1 | Susceptible |
| Oyam | Acaba | Barowor | Rainy season | *An. arabiensis* | 5 | 3 | 2 |  | Resistant |
| Oyam | Acaba | Barowor | Rainy season | *An. gambiae s.s.* | 16 | 10 | 5 | 1 | Resistant |
| Oyam | Minakulu | Bungiping | Rainy season | *An. arabiensis* | 4 | 3 |  | 1 | Susceptible |
| Oyam | Minakulu | Bungiping | Rainy season | *An. gambiae s.s.* | 93 |  | 2 | 8 | Susceptible |
| Oyam | Minakulu | Bungiping | Dry season | *An. arabiensis* | 3 | 2 |  | 1 | Susceptible |
| Oyam | Minakulu | Bungiping | Dry season | *An. gambiae s.s.* | 40 |  | 3 | 2 | Susceptible/Resistant |
| Oyam | Minakulu | Obapo | Dry season | *An. gambiae s.s.* | 7 |  | 1 | 4 | Susceptible |
| Pader | Ogom | Telela Centra | Rainy season | *An. gambiae s.s.* | 1 |  |  | 1 | Susceptible |
| Pader | Ogom | Owaleng Valley | Rainy season | *An. gambiae s.s.* | 1 |  |  | 1 | Susceptible |
