# Supplementary material for: High insecticide resistances levels in Anopheles gambiaes s.l. in northern Uganda and its relevance for future malaria control
Source: BMC Res Notes. 2020 Jul 22;13:348. doi: 10.1186/s13104-020-05193-0 (PMC7376877; doi:10.1186/s13104-020-05193-0)
Supplement: Supplementary file 3 — Additional file 3: Table S3. Knockdown summary during 60 min exposure (KD60). [file 13104_2020_5193_MOESM3_ESM.docx]

**Table S3: Knockdown summary during 60 minutes exposure (KD60)**

| **Site code** | **Insecticide** | **No. of knocked down mosquitoes** | | | | | | | **Total Exposed** | **KT50** | **KD60** |
| --- | --- | --- | --- | --- | --- | --- | --- | --- | --- | --- | --- |
|  |  | 10 | 15 | 20 | 30 | 40 | 50 | 60 |  | **(min)** | **(%)** |
| Gulu | Deltamethrin | 0 | 3 | 5 | 12 | 19 | 23 | 27 | 100 | 76 | 27 |
| Gulu | Permethrin | 0 | 3 | 4 | 6 | 8 | 8 | 9 | 100 | 76 | 10 |
| Gulu | Bendiocarb 0.1% | 0 | 18 | 37 | 75 | 91 | 95 | 98 | 100 | 30 | 98% |
| Gulu | Malathion 5% | 4 | 27 | 45 | 97 | 100 | 100 | 100 | 100 | 30 | 100% |
