# Supplementary material for: High insecticide resistances levels in Anopheles gambiaes s.l. in northern Uganda and its relevance for future malaria control
Source: BMC Res Notes. 2020 Jul 22;13:348. doi: 10.1186/s13104-020-05193-0 (PMC7376877; doi:10.1186/s13104-020-05193-0)
Supplement: Supplementary file 4 — Additional file 4: Table S4. Percentage mortality (24 h) of An. gambiae s.l. after 60-min exposure (KD60) to bendiocarb, permethrin, deltamethrin and malathion. [file 13104_2020_5193_MOESM4_ESM.docx]

**Table S4.** Percentage mortality (24 hour) of *An. gambiae s.l.* after 60-min exposure (KD60) to bendiocarb, permethrin, deltamethrin and malathion.

|  |  |  | **Exposed** | | | **Control** | | |  |
| --- | --- | --- | --- | --- | --- | --- | --- | --- | --- |
| **Site code** | **Insecticide** | **No. of test tubes** | **Mortality** | **Total mosquitoes** | **Mortality (%)** | **Mortality** | **Total mosquitoes** | **Mortality (%)** | **KD60 (%)** |
| Gulu | Bendiocarb 0.1% | 4 | 98 | 100 | 98% | 0 | 50 | 0 | 98 |
| Gulu | Permethrin 0.75% | 4 | 6 | 100 | 6% | 0 | 50 | 0 | 10 |
| Gulu | Deltamethrin 0.05% | 4 | 5 | 100 | 5% | 0 | 50 | 0 | 27 |
| Gulu | Malathion 5% | 4 | 100 | 100 | 100% | 0 | 50 | 0 | 100 |
